# A Study on Regional GDP Forecasting Analysis Based on Radial Basis Function Neural Network with Genetic Algorithm (RBFNN-GA) for Shandong Economy

**DOI:** 10.1155/2022/8235308

**Published:** 2022-01-25

**Authors:** Qing Zhang, Abdul Rashid Abdullah, Choo Wei Chong, Mass Hareeza Ali

**Affiliations:** School of Business and Economics, Universiti Putra Malaysia, Seri Kembangan 43400, Malaysia

## Abstract

Gross domestic product (GDP) is an important indicator for determining a country's or region's economic status and development level, and it is closely linked to inflation, unemployment, and economic growth rates. These basic indicators can comprehensively and effectively reflect a country's or region's future economic development. The center of radial basis function neural network and smoothing factor to take a uniform distribution of the random radial basis function artificial neural network will be the focus of this study. This stochastic learning method is a useful addition to the existing methods for determining the center and smoothing factors of radial basis function neural networks, and it can also help the network more efficiently train. GDP forecasting is aided by the genetic algorithm radial basis neural network, which allows the government to make timely and effective macrocontrol plans based on the forecast trend of GDP in the region. This study uses the genetic algorithm radial basis, neural network model, to make judgments on the relationships contained in this sequence and compare and analyze the prediction effect and generalization ability of the model to verify the applicability of the genetic algorithm radial basis, neural network model, based on the modeling of historical data, which may contain linear and nonlinear relationships by itself, so this study uses the genetic algorithm radial basis, neural network model, to make, compare, and analyze judgments on the relationships contained in this sequence.

## 1. Introduction

As economic policies become more relevant to people's lives, economic development has become a topic of discussion, and GDP, a measure of a country's or region's economic performance and level of development, has gradually become a national focus. The country's or region's future development trends are measured and evaluated. Shandong province's total GDP has ranked third among national economic provinces for many years, and according to the most recent data, its total GDP exceeded $7 trillion for the first time in 2017. Shandong province's economic development has a solid foundation. However, the gap between it and Guangdong and Jiangsu provinces, which are ranked first and second in terms of total GDP, is widening year by year [1]. Furthermore, Shandong province's total GDP ranking remains deficient when compared to its GDP per capita level; the level of GDP per capita indicates a region's level of development; for Shandong province, as a large population province, increasing GDP per capita is always a development goal. In terms of Shandong province's total GDP growth rate, it increased by 7.4 percent in 2020 compared to 2019, but the growth rate does not have an advantage over other provinces, and the economy of Shandong province still has a lot of room for development [2].

Based on the above background, it can be seen that the study of Shandong province's economic situation is very relevant and valuable, and the appropriate forecast of Shandong province's GDP can, to a certain extent, know the future economic development in advance, to be prepared in advance, and according to the forecast timely adjustment based on the existing situation. For the total economic volume of Shandong province, the situation of small per capita can explore the factors affecting the economic development of Shandong province and the interaction of Shandong province GDP and clarify the key to the economic development of Shandong province, based on the existing key transformation so that the economy of Shandong province attains healthy and stable development. Based on the development of the three major industries in Shandong province, there is still a lot of room for improvement, and the study and discussion of the GDP of each of the three major industries are conducive to further adjustment and optimization of the industrial structure and improvement of the economic development posture of Shandong province. Radial basis function (RBF) neural network is an efficient single-hidden layer forward network, which mimics the neural network structure of local regulation and mutual coverage of sensory domains in the human brain [3]. The gray forecasting model has unique advantages for forecasting with little data, missing data, or poor information, with low requirements for original data, high short-term forecasting accuracy, and ease of model testing; however, when data are scattered or the number of forecasting periods is long, the forecasting accuracy will significantly drop; the quadratic exponential smoothing method requires less historical data and has high short-term forecasting accuracy for forecasting with little data, missing data, or poor information; however, when the data are scattered or the number of RBF neural networks is a type of forward neural network that has global approximation capability and best approximation performance, it allows to approximate any nonlinear function. It differs from the BP (backpropagation) network in terms of network structure and learning algorithm, and it partially compensates for the BP network's shortcomings. In comparison to other types of forwarding neural networks, the RBF neural network has several advantages, including a simple network structure, a deep physiological foundation, fast learning ability, and good approximation performance [4]. The radial basis function combined with the genetic algorithm (RBFNN-GA) effectively avoids local minima and improves global retrieval ability by overcoming the overlearning phenomenon. The RBFNN-GA algorithm was used to conduct predictive analysis of the Shandong economic region, which provides useful information for future economic development strategy and policy.

## 2. Related Work

There are many research methods and empirical works on GDP forecasting in China and its provinces and cities, and the main methodological models are summarized as regression analysis, time series analysis, gray forecasting, artificial neural network, and a combination of these types of models. The literature [5] used five regression models to predict GDP and compared the prediction results and robustness; the literature [6] analyzed five influencing factors such as fiscal expenditure and residential consumption that affect China's GDP and built a linear regression model based on these five factors to predict China's GDP. The gray forecasting method has gained the attention of a huge amount of researchers with a smaller sample size to get higher prediction accuracy and is widely used in socioeconomic forecasting. The literature [7] used the time series data of the GDP of Jiangsu province from 1978 to 2009 and then used the GM model to forecast, and the test results showed that the model could provide accurate forecasts. Literature [8] established a gray GM model with different dimensions and an improved method of the gray model to forecast the GDP of Anhui province in 2015. Literature [9] used Lagrangian difference and segmented linear Newtonian difference methods and two orthogonalization methods to optimize the gray forecasting model to forecast the GDP growth of Xinjiang in 2018–2019. Due to their strong nonlinear fitting ability, artificial neural network models are frequently used to forecast GDP. The literature [10] used BP neural network models to forecast Guangxi GDP and found that BP networks are more informative. The literature [11] used the stepwise regression method to preprocess the data before applying the neural network method to predict the GDP of Jilin province, resulting in more accurate predictions. To build a GDP prediction model, the literature [12] combined the improved standard particle swarm algorithm with the dynamic Elman neural network with local memory function, which yielded GDP prediction values with higher accuracy. All of the above methods have produced positive results in predicting GDP, but as technology advances, the demand for accuracy and model stability grows. As a result, combined models for prediction are becoming more common, combining the benefits of each model to improve the model's applicability and prediction accuracy. To forecast GDP, literature [13] used the ARIMA model, exponential smoothing model, and a combined forecasting model with the residual inverse method, variance inverse method, and least-squares method. Literature [14] combined the time series model and regression analysis model to analyze and forecast the GDP of Anhui province. Literature [15] used gray forecasting GM and ARIMA models to separately forecast Chinese GDP and weighted average of the forecast values to get the final result to normalize. The literature [16] combined the ARIMA model with BP neural network method to form a new combined model to forecast the GDP of Shandong, studied its principle, and tested the generalization to derive the optimal forecasting model. The literature [17] empirically tests the impact of industrial structure on overall income and growth, and after discussing the detailed results of traditional shift-share analysis, it uses dynamic panel estimation to analyze a standard growth model extended by structural variables and, based on data from 28 OECD countries, concludes that industrial structure is a macroeconomic development and growth in the 1990s The conclusion that it is an important determinant of the literature [18], and others explored the link between public expenditure and economic growth, deriving conditions under which changes in the composition of expenditure lead to stable economic growth, and, using data from 43 developing countries, found that the current increase in the share of expenditure has a positive and significant growth effect, while in contrast, the relationship between the capital component of public expenditure and per capita growth is negative, with overuse of productive expenditures. There is no benefit to be derived from the excessive use of productive expenditures.

## 3. A Study on Regional GDP Forecasting Analysis of Shandong Economy in China Based on RBFNN-GA Algorithm

### 3.1. Radial Basis Function Neural Network Algorithm Combined with Genetic Algorithm

**Figure 1:**
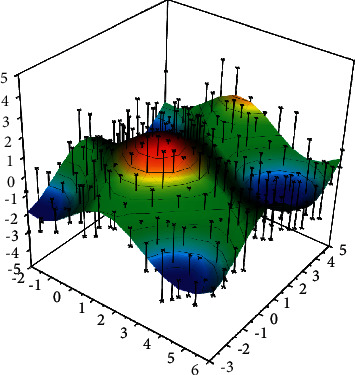
Schematic diagram of local minima.

The basic idea of the RBFNN neural network is to transform the data from a low-dimensional space to a high-dimensional space so that the data can be linearly separated after the transformation. Both RBF and BP neural networks have a pretty nonlinear fitting ability. RBFNN contains only one hidden layer and computes the parameters by the idea of local approximation, which is computationally small and fast. RBF starts by choosing *P* basis functions, each of which will correspond to a training sample, and the individual basis functions are of the form (*φ*‖*X*−*X*‖). Since the distances are radially identical, the radial basis functions get their name from this. Although both RBFNN and BP neural networks are universal approximators and belong to the same static feedforward neural network, they are always interchangeable in the same example.  Figure 1 shows a radial basis function (RBF), whereas BP is a sigmoid function. Local minima in the parameter space, as shown in Figure 1, is a common problem with traditional neural network algorithms. Because any local minima is global minima if a function is convex, many models try to use convex optimization methods, such as the well-known support vector machine algorithm, which converts the problem into a dyadic problem using KKT conditions, and the dyadic problem is a convex optimization problem, allowing the global minima to be easily found. Because of their powerful nonlinear fitting ability, RBF neural networks can map a wide range of nonlinear relationships. In contrast, most loss functions, especially in deep learning, are nonconvex, and multiple local minima can be found [19]. In addition to the function's nonconvex nature, the spatial symmetry of the neural network's weights can result in a large number of local minima, emphasizing the importance of a good optimization algorithm. To find the existence of global minima, a genetic algorithm can be used to improve the traditional radial basis neural network algorithm.

**Figure 2:**
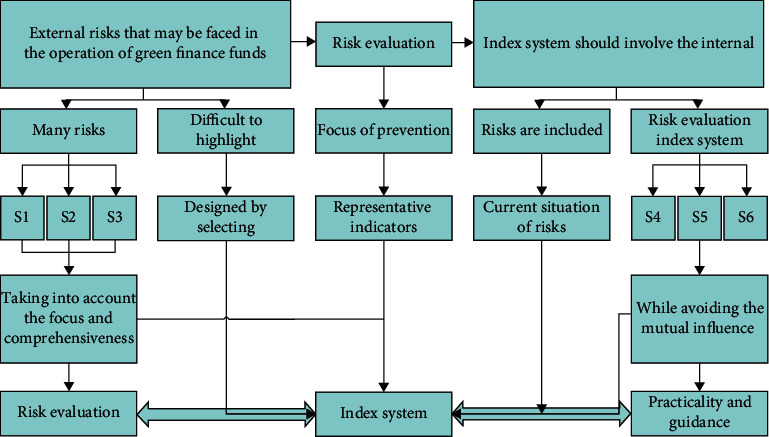
Structure of radial basis function neural network.

The mathematical model of radial basis function neural network based on genetic algorithm (radial basis function neural network-genetic algorithm, RBFNN-GA) with three-layer network structure can be represented by the following:

(1) $$\begin{array}{c} T\left(i,j\right)=\frac{\bar{g}\left(i,j\right)+\bar{g}\max}{\bar{g}\max-\bar{g}\min}+{\left|X-Y\right|}^{2}, \end{array}$$

where *X* = (*x*_1_, *x*_2_,…, *x*_*n*_) is the input vector and *Y* = (*y*_1_, *y*_2_,…, *y*_*n*_) denotes the center of the *i*th neuron node in the hidden layer; |*x*−*y*|^2^ denotes the square of the Euclidean distance between the input vector and the centroid; *g* represents the activation function, which is usually Gaussian in radial basis function neural networks; *σ* denotes the smoothing factor of the activation function of the *i*th neural node, which is used to control the width of the activation function; A denotes the weight from the hidden layer to the output layer; and *k* and *m* denote the number of nodes of the hidden layer neurons and the dimension of the input vector, respectively. The structure of the three-layer radial basis function neural warp network in this study is shown in Figure 2.

A random radial basis function neural network differs from a general radial basis neural network, and in that the centers *y*_*i*_ = (*y*_*i*1_, *y*_*i*2_,…, *y*_*i*n_) and the smoothing factor *k* of its hidden layer neuron nodes are generated in some way and need to be determined before the weights *A* of the output layer are derived by least squares. For any continuous function *f* and RBFNN-GA neural network, where *I* ∈ [0, 1] ∈ *R*, we define

(2) $$\begin{array}{c} Y=I_{nn}\cdot X_{nn}=\left(\begin{array}{ccccc} I_{11} & \ldots & I_{1j} & \ldots & I_{1n}\\ \ldots & \ldots & \ldots & \ldots & \ldots \\ I_{i1} & \ldots & I_{ij} & \ldots & \ldots \\ \ldots & \ldots & \ldots & \ldots & \ldots \\ I_{in} & \ldots & \ldots & \ldots & I_{nn} \end{array}\right)\cdot \left(\begin{array}{ccccc} x_{11} & \ldots & x_{1j} & \ldots & I_{1n}\\ \ldots & \ldots & \ldots & \ldots & \ldots \\ x_{i1} & \ldots & x_{ij} & \ldots & \ldots \\ \ldots & \ldots & \ldots & \ldots & \ldots \\ x_{in} & \ldots & \ldots & \ldots & x_{nn} \end{array}\right)=\left(\begin{array}{ccccc} y_{11} & \ldots & y_{1j} & \ldots & y_{1n}\\ \ldots & \ldots & \ldots & \ldots & \ldots \\ y_{i1} & \ldots & y_{ij} & \ldots & \ldots \\ \ldots & \ldots & \ldots & \ldots & \ldots \\ y_{in} & \ldots & \ldots & \ldots & y_{nn} \end{array}\right). \end{array}$$

The parameter *b* denotes the output threshold. Then, based on the predicted output *O* and the expected output *T*_*k*_, the prediction error can be determined as

(3) $$\begin{array}{c} b=\left(O_{k}-T_{k}\right)\cdot g\left(k\right). \end{array}$$

The update formula for the weights and thresholds is as follows:

(4) $$\begin{array}{c} \beta =\alpha_{1}-\frac{\left(\alpha_{2}+\alpha_{1}\right)\left(E_{n}^{1}+\lambda_{1}\right)}{\left(\lambda_{2}+\lambda_{1}\right)}. \end{array}$$

When the input signal of the network is the *k*th training sample *X*_*k*_, its output value of the *j*th neuron after the nonlinear transformation of the *i*-th hidden layer neuron is

(5) $$\begin{array}{c} X_{k}=T_{k}+b\sum_{i=1,j=1}^{n}X_{ij}^{2}, \end{array}$$

where *j* = 1,2,…, *J*. The basis function is generally chosen to be Green's function:

(6) $$\begin{array}{c} G\left(J\right)=j\frac{\partial \gamma }{\partial j}+\frac{1}{n}\sum_{i=1}^{n}X_{i}Y_{i}. \end{array}$$

The core concept behind RBFNN networks is that the hidden layer transforms the input data, mapping the relatively low-dimensional input into a high-dimensional space, allowing the data to be transformed from indistinguishable in the low-dimensional space to divisible in the high-dimensional space. The division of the input space into several subspaces in hyperspherical form is the geometric core of RBFNN neural networks. Theoretically, it can be argued in this study that as long as there are enough hidden layer nodes, the RBF network can always achieve an infinite approximation to any arbitrary continuous function, regardless of how small the error is set. This study also shows that two-parameter weights in the formula and the center of the radial basis function have yet to be determined and will be determined through network training. The random selection of some parameters in radial basis function neural networks is a useful addition to the existing methods for determining the center and smoothing factor of these networks. The centers of radial basis functions are frequently calculated using clustering methods (*K*-means clustering, fuzzy *C*-means clustering, and principal component clustering methods) and other methods, similar to some fuzzy modeling technologies [20, 21]. The gradient descent method is frequently used to train the weights of the hidden layer to the output layer [22]. When the RBFNN-GA neural network has a very large number of hidden layers, it can be used to approximate any *M*-element continuous function with arbitrary accuracy, and the network can always be trained to find a corresponding set of weights to make the best approximation of the unknown nonlinear mapping relation *f*(−) between the input and output. A regularized network is described in terms of the interpolation problem as complete interpolation, i.e., finding a hypersurface such that it passes through all sample points.

RBFNN-GA neural networks are nonlinear multilayer feedforward neural networks that have the same properties as multilayer perceptrons, with the exception that they are both general approximators, meaning that any multilayer perceptron can be replaced by an RBFNN-GA neural network, and any RBFNN-GA neural network can find a multilayer perceptron that performs the same function as it. However, there are some distinctions between the two networks.

1. In terms of network structure, RBF neural networks have only one hidden layer, unlike multilayer perceptrons, which can have one or more hidden layers.
2. In a multilayer perceptron, the implicit layer neuron model is identical to its output layer neuron model; in an RBFNN-GA neural network, the two models are quite different and the two models do not play the same role in the network.
3. There can be linear or nonlinear implicit and output layers between multilayer perceptrons, and when they solve pattern classification problems, they generally choose nonlinear mapping relations, and if they are solving nonlinear regression problems, the output generally chooses linear relations, and it is different in RBFNN-GA neural networks, where the implicit layer is a nonlinear mapping relation while the output layer is a linear mapping relation.
4. The excitation function of the RBFNN-GA neural network hidden layer neuron calculates the Euclidean distance between the input vector and the center, while the excitation function of the multilayer perceptron hidden layer neuron calculates the inner product between the input unit and its connection weights.
5. The RBFNN-GA neural network uses a local exponentially decaying nonlinear function to make a local approximation of the nonlinear input-output mapping, while the multilayer perceptron is a global approximation of a nonlinear mapping. To put it another way, the multilayer perceptron requires many more parameters than the RBFNN-GA neural network in order to approximate the nonlinear input-output mapping with the same accuracy.

### 3.2. Application of RBFNN-GA Algorithm to GDP Forecasting and Analysis Studies

According to the literature and various theoretical proofs, the combined model's prediction effect can usually improve the single model to some extent. Because it is difficult to tell whether time series is linear or nonlinear in real-world applications based on time series modeling, a single model is often used for one of the mappings, whereas the combined forecasting method described in this study targets complex mapping relationships and can tap both linear and nonlinear features of the series. The rising trend of GDP in Shandong province is influenced by many factors and is a nonstationary series, so its series is judged to have both linear and nonlinear characteristics. The GA model is frequently used to model linear relationships, while the RBFNN neural network is used to mine the series' nonlinear relationships. Model compression techniques and scaling were combined with optimization goals to achieve an automatic selection of model compression techniques for various networks and task requirements. The combination of these two forecasting methods fully exploits their benefits and compensates for the single model's single mapping relationship flaw. Most of the data features of the GDP series in Shandong province can be mined using a combination of RBFNN and GA neural network, and the prediction accuracy of a single model can be improved.

The network structure of the RBFNN neural network algorithm is determined, and its training process is mainly divided into two parts: first is the forward propagation of information, which means that the information is input from the input layer, after the nonlinear mapping of neurons in the hidden layer, and passed into the output layer to output the result [23]. The second is error backward propagation, which refers to the error between the output result of the output layer and the true value by judging the error, and if it exceeds the range of the preset error, the error signal is gradually passed backward, while adjusting the weights and thresholds of each hidden layer passed through, so that the adjusted parameter model makes the output error decrease along the gradient direction. After repeatedly fixing the model after the parameters until the prediction error is controlled within the set range, the learning process of the network is ended and the weights and thresholds of each layer corresponding to the minimum mean square error are obtained, i.e., the nonlinear mapping relationship of the model is determined.

The compression algorithm is improved by studying three compression techniques, namely quantization, sparse, and cropping, to realize the compression of different models with different network structures at different scales of model compression. The experiments compare the models' load characteristics under various compression methods. The differences in performance between the various methods are tracked and analyzed. Finally, reasonable model compression and scaling techniques are demonstrated. By selecting a reasonable model compression technique and ratio, the model size, inference time, and energy consumption can be effectively reduced, and the performance of different compression methods in different network structures varies. The best compression method is determined by the target neural network's structure and the optimization constraints. The best compression method is determined by the target neural network's structure and the optimization constraints.

**Figure 3:**
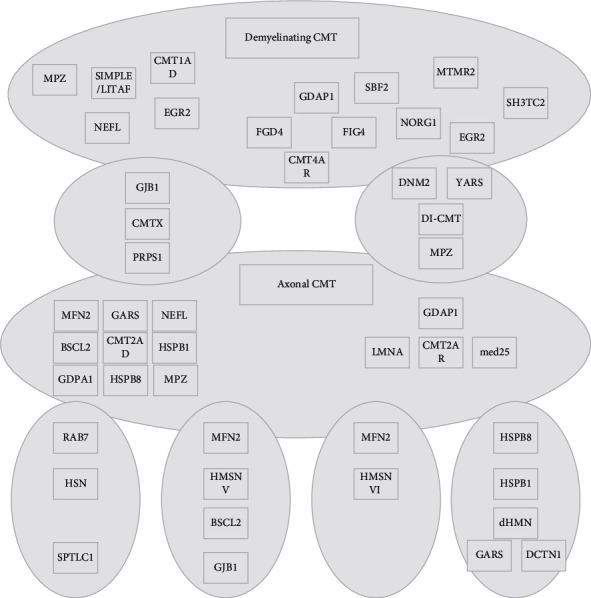
Flow chart of the combined model prediction.

Combining the two algorithms of RBFNN and GA neural network, the main principles of the combined prediction model are as follows: (1) the GA model predicts the linear part of GDP in Shandong province; because the GA model builds a linear regression of the current data about its lagged data, so this study will apply the prediction result of the GA model as the linear part of the combined model prediction. The residual of the RBFNN model prediction, i.e., the white noise, as the part outside the linear part of the series, no longer has the value information of linear prediction, so it is hoped that the residual value of white noise can be extracted using nonlinear prediction. (2). RBFNN neural network model trains the white noise series and extracts the nonlinear features in the GDP series, mentions the residual series predicted by the GA model, and builds the RBFNN neural network model after the matrix transformation and normalization process. The predicted value of this model is used as the predicted value of the nonlinear part of the time series, i.e., the nonlinear information of the extracted time series. (3) The fitted value of the GA model is added to the predicted residual value of the RBFNN neural network and the obtained predicted value, which is the predicted value of the combined model proposed in this study. The specific steps are shown in Figure 3.

The variance of each variable in the model can be decomposed according to certain criteria, and the criterion of variance decomposition is to decompose the variance of the variable according to the proportional size of the influence of each random disturbance term on the variable, and the variance decomposition studies the proportion of the influence of the orthogonalized residuals on the mean squared error (MSE) of the prediction, so it can be seen that the variance decomposition studies the proportion of the influence of the orthogon. Because the variance decomposition yields the proportion of shocks' impact on variable change, the relative magnitude of each perturbation term's impact on the variables can be determined, and thus, the relative importance of each perturbation term on a variable can be determined. The sum of the percentage contributions of all orthogonalized disturbances to a variable's effect is 100 percent for its mean square error. The vast majority of time series in socioeconomic phenomena are nonstationary time series, as is well known. In order to convert the slowly varying trend terms, periodic seasonal terms, and smooth random noise terms in nonstationary time series into stationary time series, various data primitive transformations and more processing methods must be used in modeling. This can then be transformed into analysis modeling. As a result, transforming the time series into a smooth time series and ensuring that the trend information is extracted adequately become critical. When dealing with nonsmooth time series, a variety of treatments can be considered to smooth them out.

With the RBFNN algorithm, stochastic radial basis function neural networks have been shown to have fast learning ability and powerful function approximation capability, both in theory and practice. However, due to the random selection (uniform distribution) of the centers and smoothing factors of the radial basis functions in the network, it is easy to cause the output matrix of the hidden layer to be irreversible and even pathological. This leads to the instability of the solution when solving the output layer weights, which affects the stability of the RBFNN algorithm and the performance of the random radial basis function neural network. More importantly, the stochastic radial basis function neural network solving the output layer weights by the RBFNN algorithm does not avoid the overfitting phenomenon and usually performs very well in the training set but poorly in the test set. It is found that the above problem can be effectively avoided by model regularization, i.e., adding the *l* penalty term to the original model.

**Figure 4:**
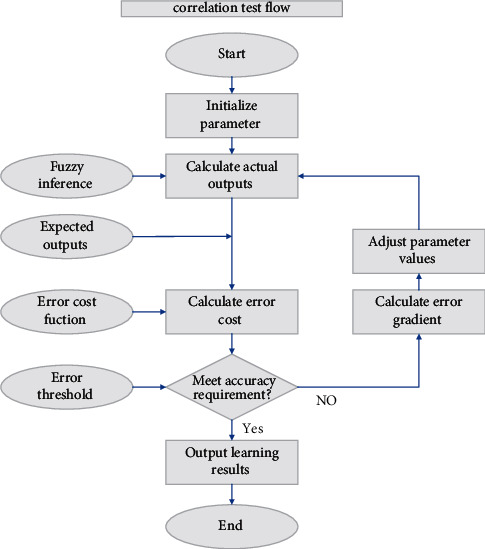
Flow chart of correlation test.

We need to preprocess the data before we can conduct the variable selection analysis, and the first step is to run a correlation test, as shown in Figure 4. The correlation coefficient between multiple sets of explanatory variables is above 0.8, and the multicollinearity between variables is serious, so it will lead to instability and difficulty in explaining the prediction model later, so in this step, we use the Lasso method to process for variable selection and solve the multicollinearity problem [24]. To free the model from the constraints of the explanatory variables' units and sizes, the data must be transformed into dimensionless, or normalized, form. Subsequently, we use the Glenmont package in *R* to implement the Lasso model for various *X* values. We can see how the model's coefficients change in the following diagram, and then, we use the cross-validation (CV) method to select the best parameter values and thus determine the variable selection results. Total energy supply and total disposable income per capita are the final economic explanatory variables we choose as the most relevant to Shandong province's GDP. This also demonstrates that Shandong province's GDP is highly correlated with agro-industry, indicating that the province's GDP is dominated by primary and secondary industries. Chemical, machinery, textile, metallurgy, and other energy-based industries account for the majority of Shandong province's main business income. Energy and raw material industries account for more than 40% of Shandong province's GDP, indicating that industrial agriculture remains the province's main contributor, and the development of tertiary industries, such as commerce, finance, and transportation and new industries such as the internet industry, is still in its infancy.

## 4. Experimental Verification and Conclusions

**Figure 5:**
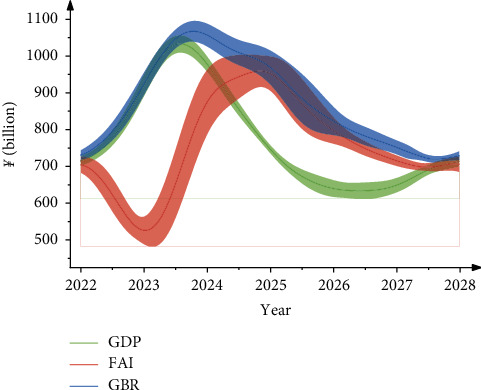
Economic aggregates projected by RBFNN-GA model for the next seven years in Shandong.

Figure 5 depicts the total economic volume of Shandong province over the next seven years, as predicted by the RBFNN-GA algorithm. It is expected that the total economic volume of Shandong province over the next seven years will continue to show an upward trend and grow at a high rate, with average annual growth rates of 3.98 percent, 5.63 percent, 6.39 percent, and 6.97 percent, respectively. GDP growth over the next five years is expected to be 7.63 percent on average, reaching 1,134.2 billion by 2025; GDP growth over the next five years is essentially the same as the growth rate in 2019–2020, is essentially consistent with the growth rate since 2015, and will remain at a higher level than in the previous 50 years. The average annual growth rate of total annual fixed-asset investment (FAI) over the next five years, 2022–2026, is forecasted to be 4.79 percent, reaching $801.95 billion in 2025; the growth rate of a total fixed-asset investment over the next five years is slightly higher than the growth rate in 2019–2020, but significantly lower than the growth rate since 2010, and will remain moderate and steady. Over the next five years, 2022–2026, the annual average growth rate of annual general public budget revenue (GBR) is expected to be 5.21 percent, reaching 947.8 billion by 2025. The general public budget revenue growth rate over the next five years is slightly higher than the rate in 2019–2020, which is largely consistent with the growth rate since 2010 and will annually increase. The initial individual's efficiency curve is the lowest, followed by the individual with the best head, and finally the individual with the best efficiency. The growth spokes of total fixed-asset investment and general public budget revenue are smaller when compared to GDP and total retail sales of consumer goods.

**Figure 6:**
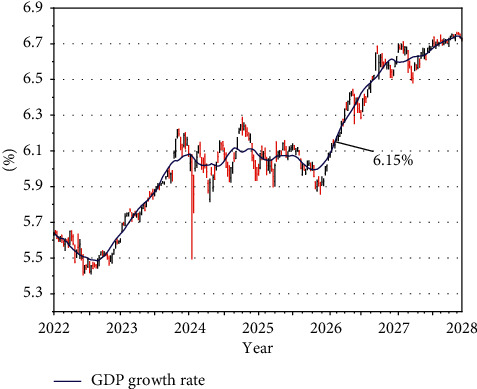
RBFNN-GA model forecasts the GDP growth rate in Shandong for the next five years.

As shown in Figure 6, in the next five years, the regional GDP growth rate is expected to increase and then decrease, but the GDP growth rate does not change much, generally located in the range of 6.3%–6.8%. It can be seen that the economy of Shandong province has passed the stage of high growth, the growth rate has become slower and entered the stage of steady progress, the regional GDP will maintain moderate growth, at this time, the total regional production increased, not only the level of economic development but also the inflation rate is small, and this level of GDP growth rate is conducive to achieve high-quality economic development. Combined with the economic development history of Shandong province, Shandong province should implement a stable and progressive fiscal policy to improve the quality of economic development while stimulating the growth of economic aggregates.

**Figure 7:**
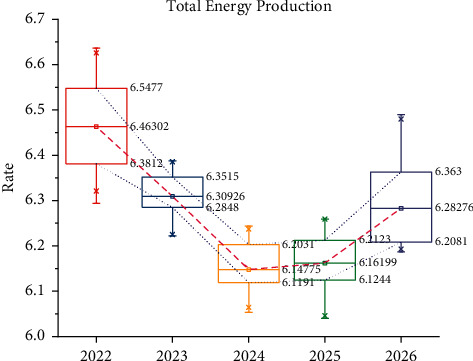
RBFNN-GA model projected changes in total energy production in Shandong over the next five years.

Figure 7 shows the change in total energy production in Shandong province over the next five years as predicted by the RBFNN-GA model. The total energy production in Shandong province is expected to decline in the next five years, with an average annual growth rate of −3.98%. The average annual growth rate of total energy production in the five years of 2022–2026 is expected to be −3.25% and will drop to 103.52 million tons of standard coal by 2025; the growth rate of energy production in the next five years is slightly higher than that in 2019–2020, but it will remain negative. Insufficient energy supply will constrain the economy and reduce the rate of economic development.

**Figure 8:**
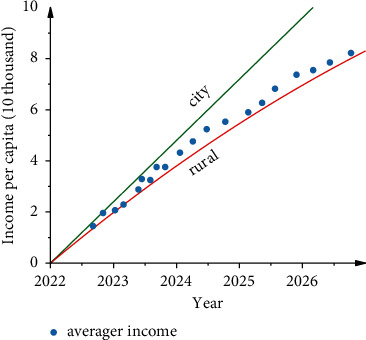
RBFNN-GA model forecasts disposable income per capita for the next five years in Shandong.

The growth rate of disposable income per capita over the next five years in Figure 8 shows that urban disposable income per capita is increasing, with annual growth rates fluctuating between 5.7 and 6.5 percent, with an average annual growth rate of 6.13 percent; rural disposable income per capita is increasing, with annual growth rates fluctuating between 7.2 and 7.9 percent, with an average annual growth rate of 6.43 percent. Both growth rates are expected to be smaller than those seen since 2005, and the difference between the two is small and consistent. In terms of growth volume, although the difference between the growth rates of per capita disposable income of rural and urban residents is not large, or even slightly higher than that of urban areas, the average annual growth of per capita disposable income of urban residents is expected to be RMB 3,145 per person, while the average annual growth of per capita disposable income of rural residents is only RMB 1,403 per person, less than half of that of urban areas. The total economic volume of Shandong province is expected to continue to show an upward trend in the next seven years, maintaining steady growth at a high rate. In absolute terms, by 2025, the per capita disposable income of urban residents is expected to reach 63,146 yuan/person (a year), but only 27,893 yuan/person (a year) in rural areas, with the absolute difference in per capita disposable income increasing from 25,093 yuan/person to 35,253 yuan/person. In relative terms, the income ratio between urban and rural residents will decrease from 2.4873 in 2018 to 1.9758 in 2025, with a slight reduction in the relative gap.

Currently, Shandong province's total economic output is at the forefront of the country, with total regional GDP in 2018 being the third highest in the country, GDP per capita the seventh highest in the country, and disposable income per capita in the first three quarters of 2019 ranked eighth in the country and will largely remain at this level over the next six years. In terms of growth, the projected GDP growth rate of 5.8% in Shandong province is not much different from the projected national GDP growth rate of about 4.7%, which is slightly lower in comparison; the per capita disposable income, especially the per capita disposable income of urban residents, will exceed the 50,000 yuan (/year) mark in 2022, and the per capita disposable income of rural residents will also exceed 20,000 yuan (/year) in the same year.

### 4.1. To Ensure Faster Economic Development and Improve the Quality of Economic Development

We should pursue progress while maintaining stability, improve the quality of economic development while stimulating economic output growth, and insist on promoting high-quality development as a general development strategy. To begin, we must vigorously develop high-tech industries and new industries, support the development of new services industries and promote development with “NEW,” and take the early zone for the conversion of old and new dynamics and the pilot. We will also encourage the growth of innovation-driven digital intelligence, and new trade patterns, the development of marine industries, local economic cooperation between China, Japan, and Korea, and the rapid economic development of coastal cities. Second, sustained and steady growth should be ensured in total retail sales of consumer goods and fixed asset investment and growth should be promoted in line with demand by lowering fees and taxes and increasing government transfer payments. Finally, secondary energy conversion rates should be improved, new energy development should be accelerated, old kinetic energy should be replaced with new kinetic energy, and adequate energy supply should be ensured for industrial development.

### 4.2. Increase Agricultural Output and Promote Coordinated Urban and Rural Development

The data on per capita disposable income show that the gap between urban and rural residents' living standards is wide and unlikely to significantly narrow in the future. To begin, we should concentrate on the three rural areas, develop new agriculture, build new rural areas, and establish a model of rural revitalization in Qilu; second, we should promote village mergers and land transfers and develop large-scale agriculture and intensive agriculture; third, we should build high-standard farmland and develop green agriculture; fourth, we should establish agricultural science and technology companies, build high-tech agricultural demonstration parks, and accelerate the construction of high-tech agricultural demonstration parks. Second, in order to build beautiful villages, rural infrastructure should be strengthened, and the road hardening and toilet revolution should be accelerated. Finally, the urbanization of rural areas and the construction of small towns should be accelerated in two ways: first, the economic strength of counties, districts, and towns should be improved to close the gap between urban and rural areas in the province, and second, smart communities should be created and digital technology should be used to serve community management.

### 4.3. Pay Attention to the Spiritual Needs of the Masses and Enrich the Cultural Life of the Residents

The standard of living of the people has been greatly improved, and their concern for the world of design has been greatly increased. We should strengthen cultural construction, enrich cultural life, and improve the quality of life in the following ways: first, through building cultural communities, cultural squares, recreational clubs, and hosting cultural tours, to involve the masses and enrich the cultural life of the people; second, through activities such as culture in the community and culture in the countryside and through cultural storytelling and watching movies, etc., to promote local culture and positive mainstream culture to the masses, to improve the cultural literacy of the people, to promote the integration of culture and technology, to create a digital platform for cultural resources, a platform for the transformation of culture and technology, etc., to cultivate new cultural industries and enhance the people's sense of cultural access, to promote the development of culture and sports, such as hosting sports events, organizing national health games, sports, and cultural festivals, etc., and to find new ways to disseminate regional culture and popularize culture in daily exercise.

For future economic development, we recommend enhancing the development of the tertiary industry on the one hand and developing the service industry on the other hand. According to recent national economic data, the service industry's scale is growing, its quality and efficiency are greatly improved, and new industries and business models are emerging. Currently, the service industry in Shandong province requires further development. The service industry has the potential to bring a variety of new economic growth points, such as productive services, in the fine development of promising financial service industries of private fundraising, cultural tourism industry, internet economic industries such as big data, cloud computing, artificial intelligence, e-commerce, and so on. Simultaneously, the growth of the service sector can help to improve and support the current Shandong province economy, which is dominated by manufacturing and agriculture industries. In light of the new economic situation and the impact of the Sino-US trade war, it is more important than ever to fully tap domestic demand and to constantly activate the development of private enterprises, and small and microenterprises to generate new economic growth points. On the other hand, in light of the current economic situation and the escalation of difficulties, we cannot dismiss the importance of the state-owned economy, which has a significant impact on the economy of Shandong province. The Shandong economy state-owned system, which has a high starting point, high level, and high-quality state-owned enterprises, should be accelerated for further development. To stimulate market players and increase market-oriented reform, the upgrading and transformation of enterprises should be sped up that are not keeping up with market development. Simultaneously, increased support for scientific research undertakings is recommended; scientific and technological innovation can significantly improve productivity, more high speed, and high quality to enhance economic growth; not only the development of applied disciplines can be transformed into productivity, but also in the current situation, the development of basic disciplines is more important and can bring more critical core technological breakthroughs. It is suggested that spending on scientific research should be increased, that the construction of “double first-class” schools and disciplines should be strengthened, and that preferential policies for attracting talents, such as subsidies for settling talents and subsidies for high-end talents, should be increased.

## 5. Conclusions

This study completes the RBFNN-GA model's GDP prediction in Shandong province, and the results show that the RBFNN-GA model's prediction accuracy with input variables is higher than the univariate RBFNN model, and the RBFNN model's stability is also higher. To improve the accuracy of GDP prediction, this study combines the two algorithms of GA and RBFNN to create a combined model. Based on the assumption that the GA model mines the linear characteristics of the GDP series, the residual series predicted by the RBFNN model is taken as the part without linear information, and the RBFNN neural network model is built to extract the nonlinear information of this residual series. The linear part's predicted value is added to the nonlinear part's predicted value, and the resulting sum is the combined model's predicted value in this study. The way the residuals are predicted is the main difference between the improved combined model and the combinatorial model. By converting the genetic algorithm input information of both the fitted value sequence and the residual value sequence, the improved model predicts the residual value of the current period, and the value is added to the linear prediction result of the corresponding radial base model to obtain the prediction value of the improved model. After analyzing the experimental results, the improved combined model has the best prediction effect. The improved model predicts the current period's residual value by converting both the fitted and residual value sequences of the GA model into RBFNN neural network input information and then by adding the value to the linear prediction result of the corresponding RBFNN model to get the prediction value of the improved model. The improved combined model has the best forecasting effect, according to the experimental results. In this study, several more important economic indicators are chosen to provide reference opinions and strategies for Shandong's economic development policies in the next five years, including total economic volume and growth rate, energy supply, and people's living standards.

## Data Availability

The data used to support the findings of this study are available from the corresponding author upon request.
